# Supplementary material for: Hierarchical Sparse Coding of Objects in Deep Convolutional Neural Networks
Source: Front Comput Neurosci. 2020 Dec 9;14:578158. doi: 10.3389/fncom.2020.578158 (PMC7755594; doi:10.3389/fncom.2020.578158)
Supplement: Supplementary file 1 [file Data_Sheet_1.pdf]

Supplementary Material

1 Supplementary Figures

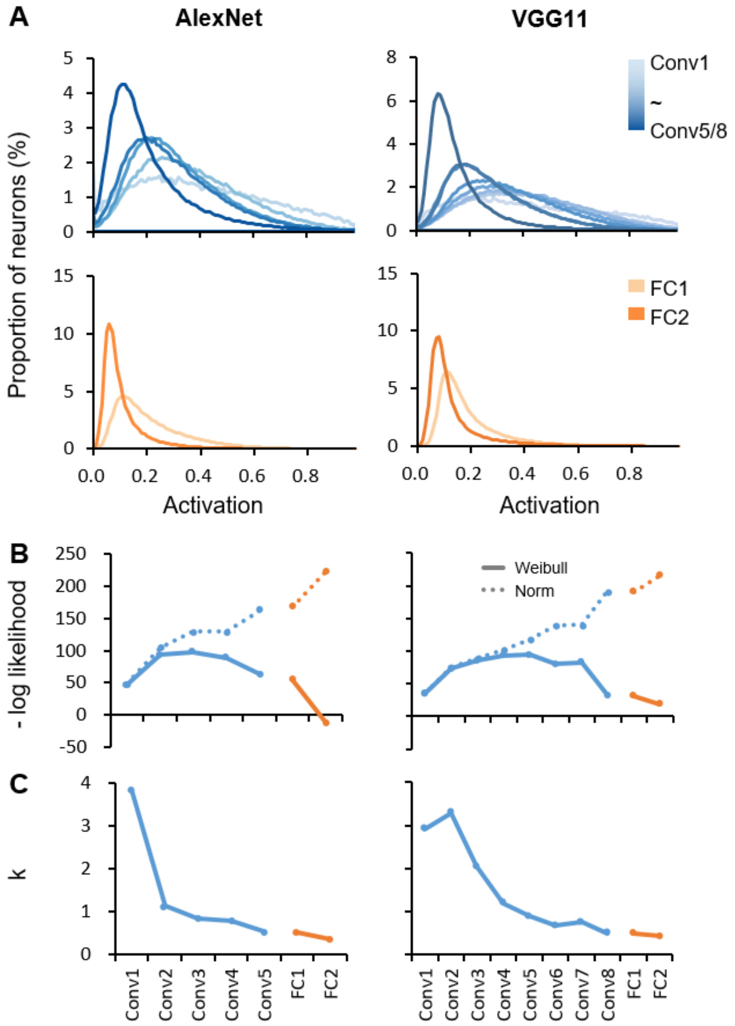

Supplementary Figure 1. Longer-tail distribution of the population activation in higher layer in DCNNs. (A) Layer-wise activation distribution of neuron population in DCNNs during object recognition. This analysis was performed on the ImageNet dataset. For each category, the activation distribution of all units in the same layer was first calculated with 100 bins between 0 to 1. Then the layer-wise activation distribution was obtained by averaging distributions over all categories. The activation distributions in higher layers showed heavier lower tails, indicating a larger number of silenced units. **(B)** Layer-wise negative log likelihood of fitting the population activation with Norm distribution and Weibull distribution. The probability density function of Norm distribution [$f(x) = \frac{e^{(-x^2/2)}}{\sqrt{2\pi}}$] is symmetric, whereas that of Weibull distribution [$f(x, k) = kx^{k-1}e^{-x^k}$] shows long-tail when k is smaller than 1. The population activation in higher layers were better fitted by Weibull distribution. **(C)** The estimated k in Weibull distribution. The estimated k became closer to 0 with the increasing layer, suggesting a longer-tail distribution in higher layer. That is, sparser population coding evolved along hierarchy.

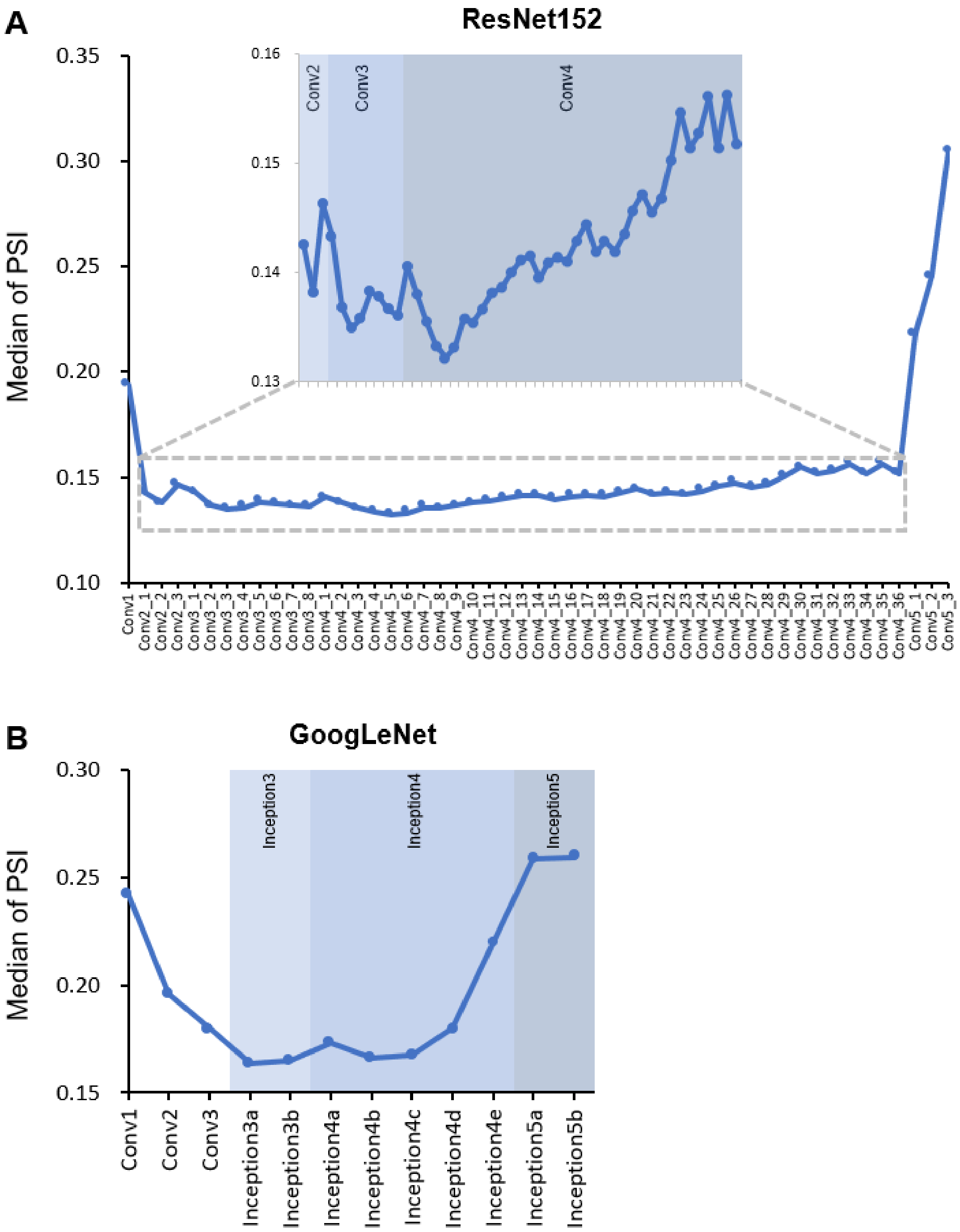

Supplementary Figure 2. Hierarchical sparse coding for object categories in ResNet152 and GoogLeNet. (A) Layer-wise population sparseness for object categories in ResNet152 (He et al., 2015). The insert is an enlarged figure of Conv2, Conv3 and Conv4. **(B)** Layer-wise population sparseness for object categories in GoogLeNet (Szegedy et al., 2014). The population sparseness was evaluated using the PSI for each object category from the ImageNet dataset (1,000 categories) in each layer separately. In both DCNNs, the median PSI of each layer showed a trend of increase along the hierarchy with an initial dip.

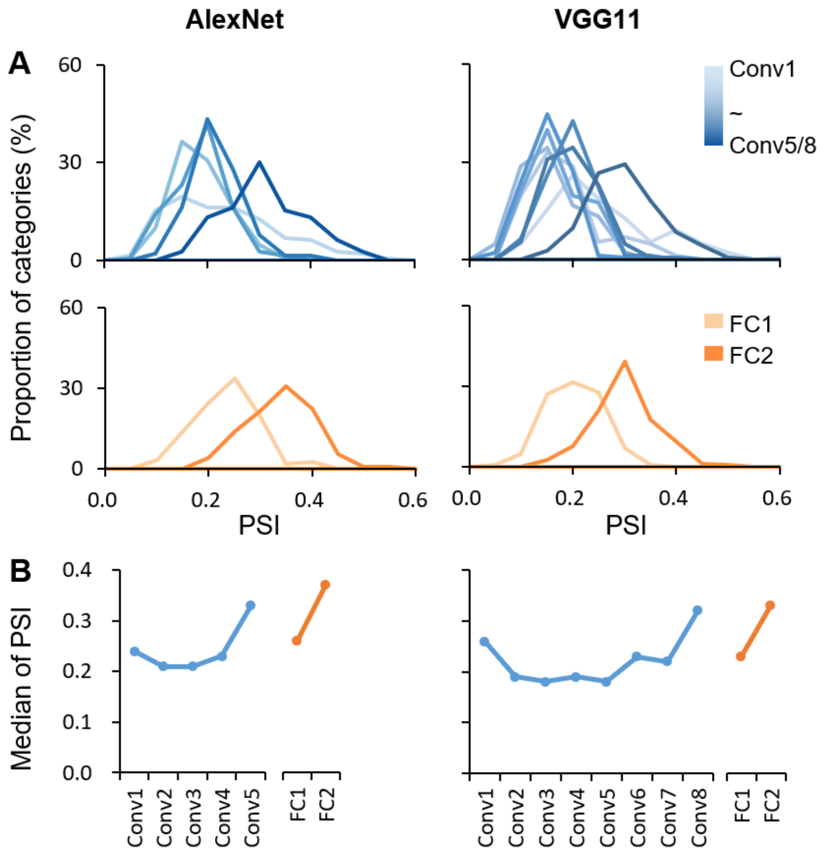

Supplementary Figure 3. Hierarchically sparse coding for object categories in DCNNs on Caltech256 dataset. (A) Layer-wise PSI distribution for objects in DCNNs on Caltech 256 dataset. Note that 113 categories among the 256 categories were excluded because of their overlap with the ImageNet dataset; therefore, the remaining 143 categories were used for validation. **(B)** Median of PSI for each layer. The median of PSI in general increased along Conv and FC layers respectively. A significant tendency was found for PSI across all layers (AlexNet: Kendall's tau = .35, $p < .001$; VGG11: Kendall's tau = .25, $p < .001$), which is consistent with the results found on ImageNet dataset as in Fig. 1.

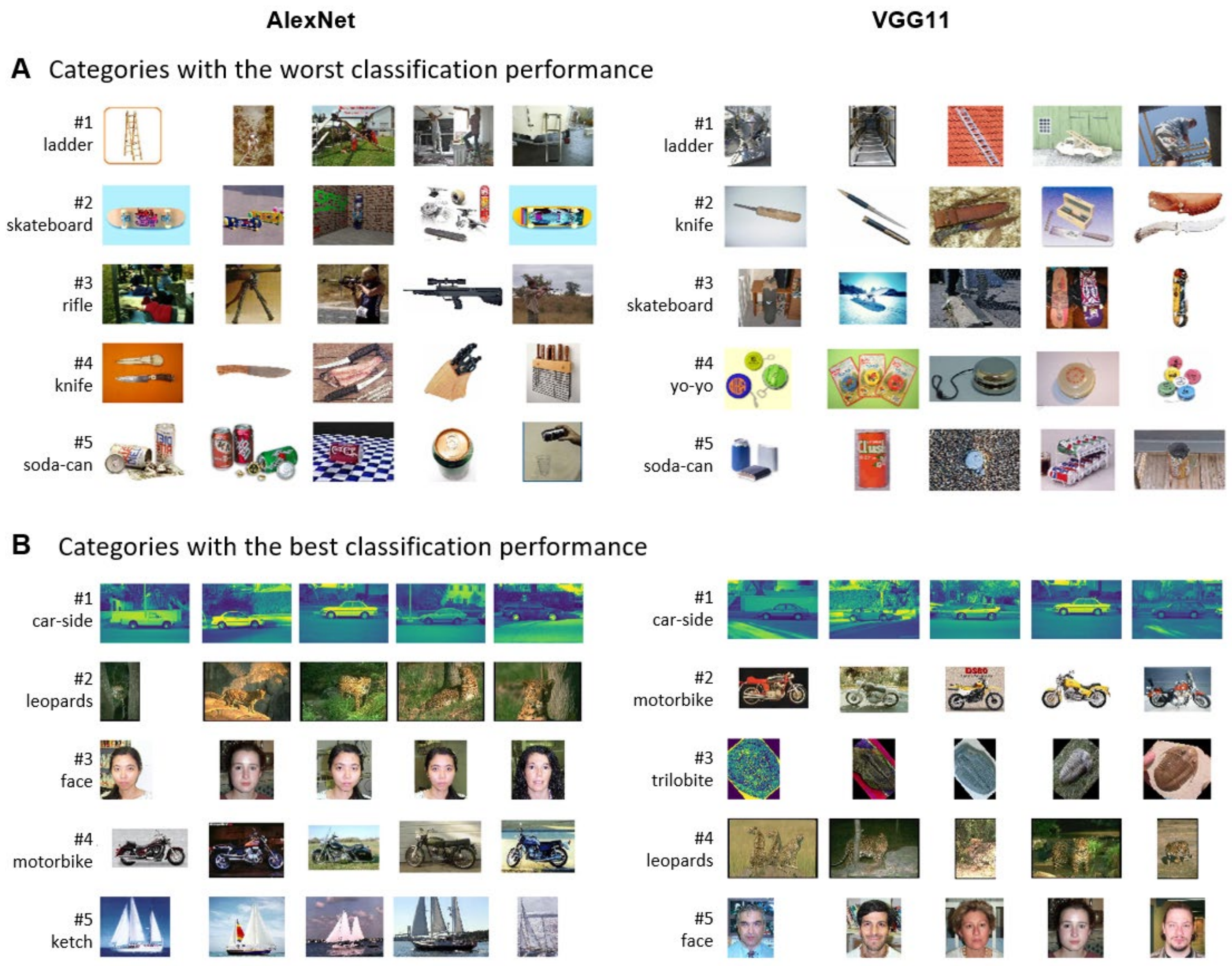

Supplementary Figure 4. Example stimuli from object categories with the best or the worst classification performance of DCNNs. The classification performance of AlexNet and VGG11 was estimated on the Caltech256 classification task using the activation from FC2. **(A)** Example stimuli from object categories with the worst five classification performance. **(B)** Example stimuli from object categories with the best five classification performance.

Reference

He, K., Zhang, X., Ren, S., and Sun, J. (2015). Deep Residual Learning for Image Recognition. *arXiv:1512.03385 [cs]*. Available at: http://arxiv.org/abs/1512.03385 [Accessed October 29, 2020].

Szegedy, C., Liu, W., Jia, Y., Sermanet, P., Reed, S., Anguelov, D., et al. (2014). Going Deeper with Convolutions. *arXiv:1409.4842 [cs]*. Available at: http://arxiv.org/abs/1409.4842 [Accessed October 29, 2020].
